# Married Women’s Attitude toward Intimate Partner Violence Is Influenced by Exposure to Media: A Population-Based Spatial Study in Bangladesh

**DOI:** 10.3390/ijerph19063447

**Published:** 2022-03-15

**Authors:** Jahar Bhowmik, Raaj Kishore Biswas

**Affiliations:** 1 Department of Health Science and Biostatistics, Swinburne University of Technology, Melbourne, VIC 3122, Australia; 2 Transport and Road Safety Research Centre, School of Aviation, University of New South Wales, Sydney, NSW 2052, Australia; raajkishore.biswas@unsw.edu.au

**Keywords:** intimate partner violence, attitude, exposure to media, low- and middle-income country, Multiple Indicator Cluster Survey, cross-sectional, binary logistic regression

## Abstract

This study estimated the attitudes of women toward accepting IPV at district level in Bangladesh and examined its relationship with sociodemographic predictors including exposure to media (e.g., newspaper, radio and television) using the Multiple Indicator Cluster Survey-2019 with a sample of 63,689 women. Around 25.6% women accepted IPV that geographically varied from 1.78% (Pirojpur) to 57.14% (Kurigram). Women regularly exposed to media were 17% less likely to accept IPV. Attitude toward accepting IPV was found to be higher among the illiterate women in disadvantaged circumstances, patriotically from poorer households living in remote areas, which suggest that planned interventions are needed for this vulnerable group of women to improve their living status by providing access to education and media. Further research is necessary to assess the impact of women’s empowerment on their attitude toward acceptance of IPV.

## 1. Introduction

According to World Health Organization (WHO), intimate partner violence (IPV) is a harmful behaviour in intimate relationships resulting from psychological abuse, physical violence, or sexual coercion [1,2]. IPV against women is one of the most common forms of violence, which is a global human right and public health concern [3,4]. In recent years, monitoring on violence against women has increased through nationwide surveys such as the Demographic and Health Survey (DHS) and the Multiple Indicator Cluster Survey (MICS). According to the WHO, the lifetime prevalence of physical or sexual violence ranges between 15% and 71% worldwide, with the lowest rates reported for Japan and the highest for Ethiopia, Peru, and Bangladesh [5]. The prevalence of IPV against women is on average 30% globally, and much higher in South-East Asian (SEA) region [6].

Bangladesh is one of the low- and middle-income countries (LMICs) in the SEA region that has one of the highest prevalence rates of IPV, ranging from 55% to 95% across 64 districts for ever married women [2,7,8]. Bangladesh is a culturally conservative country in the SEA region where IPV is typically a neglected public health issue. One of the vital indicators to achieve the Sustainable Development Goal (SDG) 5.2 set by WHO in 2015 is reduction of IPV, which is to eliminate all forms of violence against all women and girls in the public and private spheres, including trafficking and sexual and other types of exploitation.

Many socio-demographic factors were found to be associated with the prevalence of IPV, which varied from country to country including household financial status, countrywide education level, per-capita income, and rule of law [9]. Ler et al., (2017) examined IPV among ever-married women using a nationally representative study in India and reported that the prevalence of IPV was 29% and significant associated factors of IPV were age at first marriage, parental IPV, husband’s controlling behaviours, and husband’s consumption of alcohol. Studies on both developed and developing countries observed some common socio-demographic factors associated with IPV such as age, race, gender and genetic predisposition, mental disorders, abusive childhood, exposure to paternal violence, education and income [8,10,11,12,13,14].

Past studies well-demonstrated that victims of IPV encounter much higher rates of mental health conditions including anxiety and depression, post-traumatic stress disorder, drug and alcohol abuse, sexually transmitted infections, and suicide [11,15,16,17,18]. A systematic review conducted by [11] observed that mental health problems including mood and anxiety disorders, substance and drug use disorder, and suicidal attempts were the potential risk factors for physical IPV in female victims. This study also reported that poverty, job instability, and lack of social support can increase the risk of depression and anxiety in female victims of physical IPV.

Women are often vulnerable due to socio-economic disadvantages, as well as some cultural and religious restrictions imposed on them [19]. A study conducted in a southern European city by Gracia et al. [20] demonstrated that neighbourhoods with low levels of income and education and high levels of residential mobility and criminality had higher relative risk of IPV against women (IPVAW), and IPVAW is highly prevalent in disadvantaged neighbourhoods. Attitudes toward acceptance of IPV and normative cultural attitudes about IPV are the pressing factors associated with the perpetration of IPV and community responses to perpetration [21,22,23,24], especially among people living in low-sociodemographic conditions in the LMICs. Neville et al. (2004) demonstrated that women who find IPV acceptable are more likely to blame themselves for the violence and less likely to report it to family members or judicial authorities, and consequently suffer from mental health issues. Community attitudes towards IPV and its consequence can play an important role in tackling the violence [25]. People who consider IPV as a cultural norm tend not to support the victims [24,26,27,28]. In the LMICs in the SEA region and sub-Saharan Africa, the proportions of women who accept spouse-beating are 57% and 74% respectively [29,30].

During the past two decades, mass media have been used to create awareness of many public health issues, including women’s mental and physical health. Health promotion is an effective tool for promoting healthy lifestyles and preventing unhealthy behavior [31,32,33]. Media can play an important role in controlling domestic violence including IPV through awareness campaigns [34,35,36]. In a LMIC such as Bangladesh, where the public health care system has improved significantly during the last decade despite a low-functioning economy, exposure to media has been hypothesised as a vital asset for improving women’s autonomy, empowerment, and health care accessibility [37,38,39,40]. In line with this hypothesis, the current study examined the impact of exposure to media on the attitude of married women toward IPV in Bangladesh.

In LMICs, attitudinal data on IPV against women began to accumulate in the 1990s, when the surveys such as Multiple Indicator Cluster Surveys (MICS) began to ask whether wife beating was justified [13,41,42]. By evaluating 88 national surveys on lower-income countries, Yount et al. (2014) observed that justification of wife beating ranged from 4% to 90%, which was also high for Bangladesh [6,19,24,29,43]. However, there is a lack of recent large-scale population-based research on IPV among women, their attitude toward IPV, and possible actionable factors, which can be used for interventions to assist in achieving the Sustainable Development Goal 5.2 by 2030.

The aim of this study was to conduct a district-wise estimate of the proportion of women who hold the attitudes of accepting IPV against women; to assess its association with exposure to media and other sociodemographic factors; and to identify the most vulnerable cohorts through spatial analysis. In Bangladesh, national broadcasting channels (TV and radio), newspapers, and magazine regularly present information on health awareness and women’s health and wellbeing. It was hypothesized that exposure to media would be associated with women’s attitude toward accepting IPV in Bangladesh and the districts with higher acceptance rate of IPV would be the ones with having relatively lower rate of media exposure.

## 2. Materials and Methods

The aims of this study were to estimate the district-wise proportion of women holding attitudes that IPV against women is acceptable; and to investigate the association between exposure to media and attitude of accepting IPV among women in Bangladesh. Also, the final goal of this study was to identify the vulnerable cohort of women in Bangladesh through significant predictors, which can lead to actionable changes including interventions.

### 2.1. The Survey

This study analysed data collected from a large sample of women from 64 rural and urban districts in Bangladesh in the latest round six of MICS conducted in 2019. The MICS 2019 is a cross-sectional, population-based survey of Bangladeshi ever married women aged 15–49 conducted from January 19 to 1 June 2019 using a multistage, cluster sampling technique [44]. In MICS, married girls aged 15 years or above are considered as women. In Bangladesh, the new Child Marriage Restraint Bill 2017 marriage sets 18 as the legal age of marriage but with some leeway for under 18 marriages with parent consent [45]. This survey was carried out by Bangladesh Bureau of Statistics (BBS) in collaboration with UNICEF Bangladesh, as part of the Global MICS Programme. The sample for the Bangladesh MICS 2019 was designed to provide estimates for many public health indicators on children and women at the national level for 8 divisions and 64 districts. The numbers of primary sampling unit (PSU) and sampled households in the survey were 3220 and 64,400, respectively. The urban and rural areas within each district were identified as the main sampling strata and the sample of households were selected in two stages. Within each stratum, a specified number of census enumeration areas was selected systematically with probability proportional to size. After a household listing was carried out within the selected enumeration areas, a systematic sample of 20 households was drawn in each sampled PSU. Finally, data were collected from the selected households through face-to-face interviews. A more detailed description of the study design including sampling procedure and all the questionnaires used in data collection are available at http://mics.unicef.org/tools?round=mics6 (accessed: 10 December 2021).

### 2.2. Variables

In this study, the outcome variable was attitude of women’s acceptance of IPV, a binary variable with woman whose attitude was in favour of acceptance of IPV (yes) and against IPV (no). Attitude toward acceptance of IPV against women was assessed by a single set of fixed response yes/no questions: If a husband is justified in hitting or beating his wife in any of the following circumstances (1) she goes out without telling him, (2) she neglects the children, (3) she argues with him, (4) she refuses to have sex with him, & (5) she burns the food. These survey questions were included in MICS 2019 questionnaire under the heading ‘Attitude Toward Domestic Violence’ and the opinions on these questions were given by the participants during the data collection time. Attitudes accepting of IPV were categorised as ‘yes’ if at least one of the five situations had been endorsed with an affirmative answer and as ‘no’ otherwise [24,46].

According to past literature and outcomes from the pre-analysis results (missing values and consistency of variables in the surveys over the years), 10 sociodemographic factors were included in this study as explanatory variables [2,15,36]. The selected explanatory variables are age of respondents (≤25 years old versus >25 years); residence (urban, rural); division (Dhaka, Barisal, Chattagram, Khulna, Mymensingh and Rajshahi), education of both respondent and her house head (none, primary, secondary, higher); wealth index (poorest, poorest, middle, richer, richest); gender of house head (male, female); age of house head (15–34, 35–49, 50 and above); media exposer (yes, no); and mobile phone ownership (yes, no). For media exposure, if the respondent said yes to watching TV, listening to radio, reading newspaper, or used internet in the past week, she was considered as exposed to media (yes). The inbuilt household wealth index was based on asset variables using principal component analysis [47]. For model adjustment purpose, survey weights, strata, and cluster information were also extracted.

### 2.3. Statistical Analysis

Descriptive analysis was used to estimate the percentage of women’s attitude of accepting IPV. Initially a bivariate analysis was conducted where attitudes toward acceptance of IPV among women was tabulated across various factors including media exposure, age, residence status, education, wealth index, division, age of house head, gender of house head, and mobile phone ownership. These unweighted associations were tested using Chi-square tests. Following that, a Generalised Linear Model (GLM) with binary outcomes adjusting for survey cluster, strata, and weights was conducted, which provided greater scope of generalization and adjusted effect sizes. This model examined the associations between attitudes toward acceptance of IPV among women and socio-demographic variables including media exposure, age, residence status, education, wealth index, age of house head, gender of house head, and mobile phone ownership were examined. The model fitted for the outcome variable attitude toward acceptance of IPV was controlled for all the sociodemographic factors to address possible confounding effects. The attitude of accepting IPV and exposure to media among women were mapped among the 64 rural and urban districts in Bangladesh. The modelling and mapping were conducted using R-package ‘survey’ and ‘maps’ & ‘ggplot2′. All data compilations and analyses were conducted in R (3.5.0).

## 3. Results

In the MICS 2019, data from 63,689 ever married women aged between 15 and 49 years were available for analysis from initial survey response of 68,709; that is, 7.3% were missing. The women were 30 years of age on average. About 37.4% of the women were aged below 26. In terms of education, 16% of the women had no or pre-primary education, 23% had primary, 45% had secondary, and 16% had higher secondary or above education. A large majority of the women (80%) resided in rural areas. The detailed breakdown of sociodemographic factors is given in Table 1.

**Figure 1:**
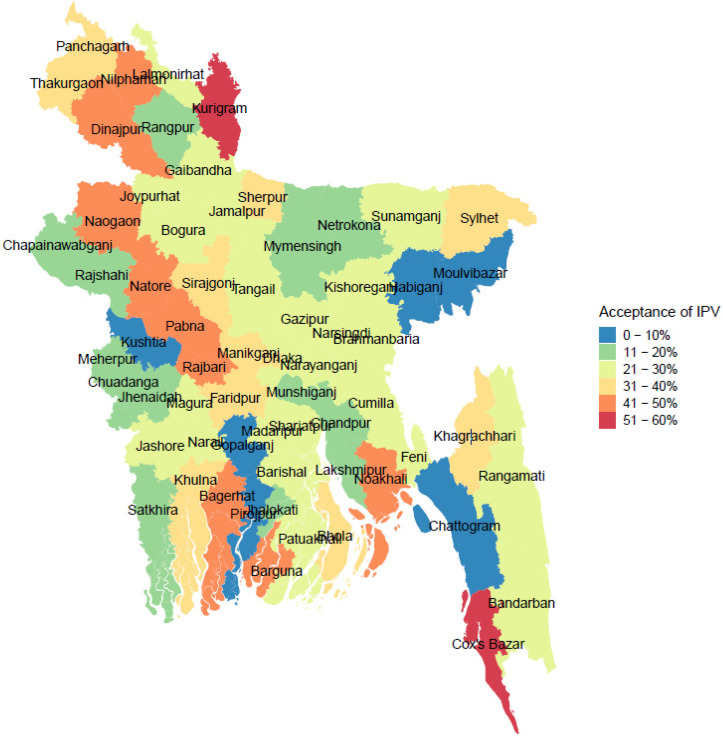
Geospatial distribution of women’s attitude toward acceptance of IPV among 64 districts in Bangladesh.

In the survey, the percentage of women having attitudes accepting of a husband beating his wife in at least one of the five situations in Bangladesh was 25.6% on average. This percentage of women’s attitude towards acceptance of IPV varied among the major districts according to their geographical location and economic status. The highest percentage of attitude among women justifying IPV was 57.14% in Kurigram and the lowest was 1.78% in Pirojpur. Figure 1 presents the mapping of women’s attitude of accepting IPV among 64 districts in Bangladesh, which shows that the percentage was higher in the northern part of the country, especially in Kurigram, Nilphamari, Dinajpur, Noagaon, Natore, Pabna, and Rajbari, and overall lower in the southern part of the country. However, some districts in the southern part of Bangladesh including Bagerhat, Barguna, Noakhali, and Cox’s Bazar also reported a higher attitude toward acceptance of IPV (Figure 1) than most other districts. Overall, the percentage of women’s attitude toward accepting of IPV is higher in the regional and peripheral districts compared to central metropolitan areas. 

**Figure 2:**
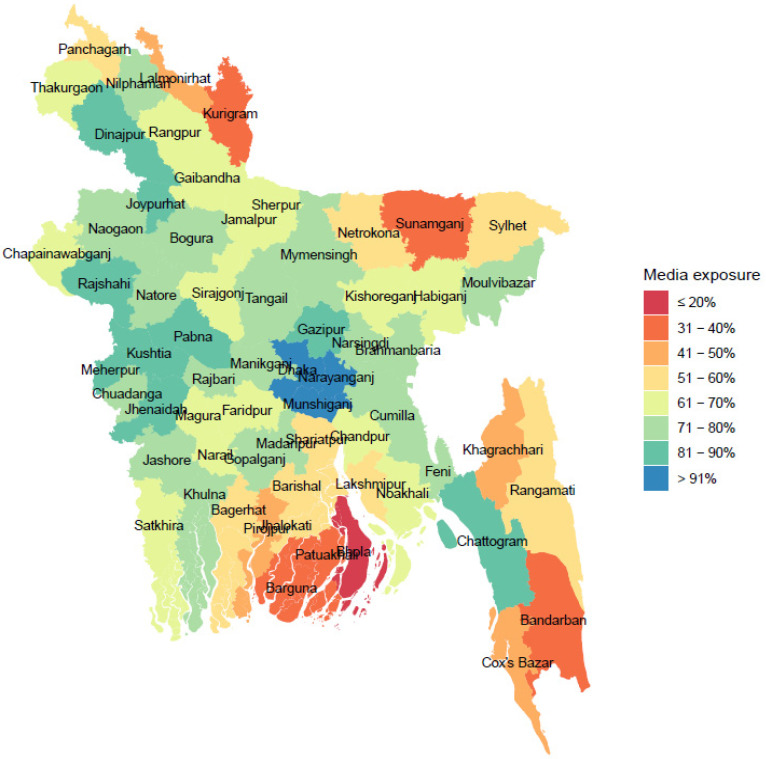
Geospatial distribution of women’s exposure to media among 64 districts in Bangladesh.

The distribution of exposure to media and its mapping among 64 districts were presented in Figure 2. The percentage of women exposed to media was highest in Dhaka (94.3%) and lowest in Bhola (28.7%). Overall, this percentage of exposure to media was higher in the urban areas and lower in the regional and remote areas. As can be seen from Figure 2, the vulnerable cohort of women were those who were living in the remote and regional districts, for example, Patuakhali, Kurigram, Sunamgong, and Bandarban had lowest exposure (<40%) to media. The results presented in Figure 1 and Figure 2 suggest that there is an association between attitude toward accepting of IPV and exposure to media among the women in Bangladesh; exposure to media mediated women’s opinion on their attitude toward accepting IPV. 

Table 2 presents bi-variate association between attitude toward accepting of IPV among women and potential sociodemographic factors. Most of the sociodemographic factors such as age, education, wealth index, residence, division, age and gender of house head, ownership of a mobile phone, and exposure to media were significantly associated with their attitude toward acceptance of IPV (*p* < 0.05). Among the women who were not exposed to media, 31.3% of them showed an attitude toward accepting of IPV, whereas for women who had exposure to media, 22.8% held this attitude. Among the eight divisions, average attitudes of accepting IPV among women was highest in Rangpur (35.8%) and lowest in Sylhet (15.8%). 

The associations between attitude toward accepting of IPV among women and potential sociodemographic factors were also tested using binary logistic regression models after adjusting for survey weights and cluster and strata-wise variations (Table 3). Exposure to media was significantly associated with women’s attitudes of accepting IPV; among the women, those who were regularly exposed to media were 17% less likely to justify IPV for any reason (AOR = 0.83; 95% CI: 0.78, 0.87, *p* < 0.001) as compared to those who were not exposed to media. Older women (>25 ys) were 27% more likely to accept any form of IPV as compared to the younger group (≤25) (AOR = 1.27; 95% CI: 1.20, 1.34; *p* < 0.001). The association between level of education and holding the attitudes of accepting IPV was significant for all three levels of education. Women with primary, secondary, and higher secondary or above education were 8%, 33%, and 60% less likely to accept IPV respectively compared to the illiterate women (Table 3). Also, women from households where the house head had primary, secondary, and higher secondary or above education were 9%, 12%, and 23% less likely to hold the attitude toward accepting of IPV, respectively, compared to households with uneducated house heads. The area residence was associated with attitudes of accepting IPV; women living in urban areas were 9% more likely to justify IPV (AOR = 1.09; 95% CI: 1.01, 1.18, *p* < 0.001) compared to women living in rural areas. 

Gender of the house head was significantly associated with women’s attitude of accepting IPV; having a male house head had 17% lower likelihood of women accepting IPV (AOR = 0.83; 95% CI: 0.76, 0.90, *p* < 0.001). Economic condition of the household was associated with women’s attitudes of accepting IPV; women living in poorer, middle, richer and richest wealth index were 7%, 16%, 23%, and 41% less likely to justify IPV respectively as compared to women living in the poorest wealth index. Age of the house head had a significant impact on women’s attitudes toward accepting IPV, women living under a house head aged between 35–49 years, and 50 and above years old were 18% and 21% less likely to justify IPV, respectively, as compared to their younger counterpart (15–34 years old). The division women lived in was associated with attitudes of accepting IPV. Women living in the divisions of Chattagram, Barisal, Khulna, Mymensingh, and Sylhet were significantly less likely to support IPV than the women living in Dhaka division. However, women living in Rajshahi and Rangpur were significantly more likely to support IPV than the women living in Dhaka division. 

## 4. Discussion

The analysis revealed that on average 25.6% of the women are likely to justify IPV, which varied from 1.78% to 57.14% across the 64 diverse districts of Bangladesh. In general, the attitude toward justification of IPV among women was higher in the peripheral districts. Analysis also revealed that the proportion of exposure to media among women in Bangladesh was heterogeneously distributed among the districts ranging from 57.14% in Kurigram to 1.78% in Pirojpur. The attitude toward acceptance of IPV among women changed very little in Bangladesh over the past 5 years, with a prevalence rate from 28.7% in 2014 to 25.6% in 2019, which accentuates the importance of the current study findings.

The association between attitude toward acceptance of IPV among women and most of the selected sociodemographic factors including exposure to media was found to be significant. Consistent with the findings from previous studies, it was found that not being exposed to media, living under a male house head, residing in rural areas, belonging to the poorest quintile of wealth index, and having a low educational level were associated with greater likelihood of justifying IPV among women [19,21,29,48]. Past studies demonstrated that media campaigns are an effective awareness dissemination tool for rural and remote areas in Bangladesh where literacy rate is relatively low [38,39,49]. Media campaigns have shown great success in promoting healthy behaviours and breaking taboos, particularly in LMICs, as means of entertaining [50,51,52]. While access to media is easier in urban or metropolitan districts, reaching the vulnerable women in peripheral districts is tougher, where the traditional norms are more resilient [53]. This study revealed that women with no exposure to media were more likely to justify IPV in Bangladesh compared to their counterparts. However, mobile phone ownership was not found to be associated with attitude toward acceptance of IPV among the women. 

In households of low socioeconomic status and low literacy levels located in Asia, IPV against women is often considered as a ‘right’ of the husband or partner to ‘correct’ his wife [29]. Uneducated women from poor households are forced to financially depend on their husband/partner or in-laws, which often lead to a substandard mentality to gradually accept any forms of IPV [20,44]. Financial dependence on in-laws is a common reason for high dowry in Bangladesh, which leads to IPV (Naved & Persson; Young & Hassan). In this current study, results indicate that the attitude toward acceptance of IPV among women in the poorest section of the society was much higher than among the women in the wealthy quintiles. 

The substantial heterogeneity of the attitudes toward IPV among women across the districts and regions also appears to be associated with women’s level of education. Heise & Kotsadam found that level of education has bivariate association with the level of IPV. The more highly educated the population is, the less prevalent attitudes among women and men supportive of wife beating are [24]. Jewkes demonstrated that low levels of education are associated with higher gender-inequality and with norms about the acceptability of using violence as an approach in relationship battle and the expression of frustration or anger. Consistent with previous findings, it was revealed that younger women (below 26) were more likely to justify IPV than older women (above 25) [21,54,55]. Furthermore, women who are married off early lack the employment skills, so they rarely contribute to family income, which often leads to an inferiority complex among these women and leads to an acceptance of IPV [19,56]. Consistent with prior evidence, it was found that the acceptance rate of IPV among women with no education or below primary level education was higher than the acceptance rate among the women with primary, secondary, and higher secondary or above level of education.

Past studies demonstrated that IPV is more common in rural and remote areas than urban areas in Bangladesh [57,58,59]. In a recent study in 39 LMICs, Tran et al. (2016) found that the acceptance rates of IPV among the women living in rural areas was higher than among the women living in urban areas. The district’s neighbouring divisional cities also seemed to have had higher media access. The argument of rurality seems to be compounded with media access, which reveals that women residing in the metropolitan areas were less likely to justify IPV; however, women from urban households were found to have greater odds of justifying IPV in this study. With the increased rural–urban migration in Bangladesh and stronger presence of organisations on women rights in divisional areas, it is likely that even urban areas in peripheral districts seem to have lesser exposure to modern health amenities and gender inequality benefits [60,61,62].

Bangladesh is a conservative, religious country where women’s autonomy is limited, and the majority of the people conform to the traditional norm that women are supposed to be excluded from the household decision making process [54,62,63,64]. This culture of forcing decisions on women make them vulnerable and often obligate them to accept any treatment from partners or in-laws. Living in peripheral districts, where women rarely have the opportunity to be aware about their rights, they consider IPV as part of their life. Awareness among the women living in poverty and having no or low education through media campaigns about their health and wellbeing is one of the critical issues in improving their attitude toward acceptance of IPV, specifically to reduce regional gaps and disparities. 

This study has some limitations. The population of the study comprised only married women between 15 and 49 years of age; women who were not married and are older than 49 years of age their attitudes towards IPV might be different than those included in the study. MICS 2019 only assessed attitudes towards physical abuse but did not include any other type of violence such as mental or emotional, which limits the study to only physical IPV. Some variables (e.g., political orientation, religiosity etc.) that are known to be associated with attitudes towards IPV are not available in the MICS data. The survey data did not include any qualitative information such as background information on reasons for such opinions or social bindings regarding media access. The majority of the women that participated in the survey were from rural areas and results of this study could be influenced by this category although the representative sample size of the women from the urban area was more than 12000 and statistical modelling was done after adjusting for the weight variable. Finally, as the study was cross-sectional, any causal interpretation must be done carefully. A future study can make comparisons between the opinions of married men and married women once men’s opinions data are available. Further studies can conduct subgroup analysis to compare advantaged and disadvantaged groups to explore the effect of media among them. 

## 5. Conclusions

This study analysed data obtained from round six of MICS 2019 conducted in Bangladesh to investigate the significant predictors of women’s attitude toward acceptance of IPV by targeting exposure to media so that the vulnerable cohort of women could be predicted. The acceptance rates of IPV among women in Bangladesh varied widely across the regional and urban districts. This study reveals that the married women without exposure to media, living in the poorest households, illiterate or low level of education, married at younger age, living with men as head of the house, and living in rural or remote areas were the most vulnerable cohort who considered IPV as acceptable. These findings indicate that national level policy discussions to achieve the key SDGs of gender equity and good health and wellbeing are urgent.

Effective and structured intervention programs with community outreach might be required to achieve SDG 5.2 by 2030, which is specific to IPV. Given media engagement is an actionable issue, future studies could work on policy interventions where media campaigns through innovative outreach strategies could be used to reduce the disparities among the women living rural and urban districts and by decentralizing the district level health systems in Bangladesh. This study’s findings provide information for policy makers to obtain an overall assessment of the characteristics of the indicators of attitude toward IPV against women in Bangladesh. National level policies aiming to improve gender equity, socio-economic status, level of education, and exposure to media can reduce the prevalence of women’s attitudes toward acceptance of IPV.

## Figures and Tables

**Table 1:** Sociodemographic characteristics of the women sampled in the survey (N = 63,689).

Variables	N	Percentages
**Age**		
≤25	23,841	37
≤25	39,848	63
**Education**		
None or pre-primary	10,301	16
Primary	14,695	23
Secondary	28,560	45
Higher secondary	10,133	16
**Wealth index**		
Poorest	13,144	21
Poorer	13,240	21
Middle	13,379	21
Richer	12,984	20
Richest	10,942 (17%)	17
**Area of Residence**		
Rural	51,042 (80%)	80
Urban	12,647 (20%)	20
**Division**		
Dhaka	12,761	20
Barishal	5463	8.6
Chattagram	11,948	19
Khulna	10,038	16
Mymensingh	3303	5.2
Rajshahi	7505	12
Rangpur	7770	12
Sylhet	4901	7.7
**Age of house head**		
15–34	12,383	19
35–49	27,519	43
50 and above	23,787	37
**Gender of house head**		
Male	57,038	90
Female	6651	10
**Education of house head**		
None or pre-primary	21,406	34
Primary	17,923	28
Secondary	17,036	27
Higher secondary/above	7324	11
**Media exposure**		
No	20,577	32
Yes	43,112	68
**Mobile phone ownership**		
No	19,321	30
Yes	44,368	70

**Table 2:** Bi-variate association (unweighted) between attitude toward acceptance of IPV and the selected covariates; N = 63,689.

Variables	Attitude towards IPV [N (%)]		*p*-Value
	No	Yes	
**Age**			
≤25	18,896 (79.3)	4945 (20.7)	<0.001
>25	28,504 (71.5)	11,344 (28.5)	
**Education**			
None or pre-primary	6574 (63.8)	3727 (36.2)	<0.001
Primary	10,009 (68.1)	4686 (31.9)	
Secondary	21,991 (77.0)	6569 (23.0)	
Higher secondary	8836 (87.1)	1307 (12.9)	
**Wealth index**			
Poorest	8702 (66.2)	4442 (33.8)	<0.001
Poorer	9205 (69.5)	4035 (30.5)	
Middle	10,014 (74.8)	3365 (25.2)	
Richer	10,206 (78.6)	2778 (21.4)	
Richest	9273 (84.7)	1669 (15.3)	
**Area of Residence**			
Rural	37,572 (73.6)	13,470 (26.4)	<0.001
Urban	9828 (77.7)	2819 (22.3)	
**Division**			
Dhaka	9425 (73.9)	3336 (26.1)	<0.001
Barishal	4191 (76.7)	1272 (23.3)	
Chattagram	9114 (76.3)	2834 (23.7)	
Khulna	7927 (79.0)	2111 (21.0)	
Mymensingh	2491 (75.4)	812 (24.6)	
Rajshahi	5137 (68.4)	2368 (31.6)	
Rangpur	4988 (64.2)	2782 (35.8)	
Sylhet	4127 (84.2)	774 (15.8)	
**Age of house head**			
**15**–**34**	8992 (72.6)	3391 (27.4)	<0.001
**35**–**49**	20,245 (73.6)	7274 (26.4)	
**50 and above**	18,163 (76.4)	5624 (23.6)	
**Gender of house head**			
Male	42,185 (74.0)	14,853 (26.0)	<0.001
Female	5215 (78.4)	1436 (21.6)	
**Education of house head**			
None or pre-primary	14,866 (69.4)	6540 (30.6)	<0.001
Primary	13,118 (73.2)	4805 (26.8)	
Secondary	13,265 (77.9)	3771 (22.1)	
Higher secondary/above	6151 (84.0)	1173 (16.0)	
**Media exposure**			
No	14,129 (68.7)	6448 (31.3)	<0.001
Yes	33,271 (77.2)	9841 (22.8)	
**Mobile phone ownership**			
No	13,876 (71.8)	5445 (28.2)	<0.001
Yes	33,524 (75.6)	10,844 (24.4)	

**Table 3:** Association between socio-demographic characteristics and attitude toward acceptance of IPV among women in Bangladesh: Results (adjusted) from logistic regression models.

Variables	AOR (95 % CI)	*p*-Value
**Age** (ref: ≤25)		
>25	1.27 (1.20, 1.34)	<0.001
**Education** (ref: None or pre-primary)		
Primary	0.92 (0.86, 0.98)	<0.001
Secondary	0.67 (0.63, 0.72)	0.009
Higher secondary/above	0.40 (0.37, 0.45)	<0.001
**Wealth index** (ref: Poorest)		
Poorer	0.93 (0.87, 1.00)	<0.001
Middle	0.84 (0.78, 0.91)	0.059
Richer	0.77 (0.71, 0.84)	<0.001
Richest	0.59 (0.53, 0.66)	<0.001
**Area of Residence** (ref: Rural)		
Urban	1.09 (1.01, 1.18)	<0.001
**Division** (ref: Dhaka)		
Barishal	0.76 (0.68, 0.85)	0.0230
Chattogram	0.77 (0.71, 0.84)	<0.001
Khulna	0.73 (0.68, 0.79)	<0.001
Mymensingh	0.66 (0.57, 0.77)	<0.001
Rajshahi	1.18 (1.09, 1.28)	<0.001
Rangpur	1.25 (1.16, 1.36)	<0.001
Sylhet	0.58 (0.51, 0.66)	<0.001
**Age of house head** (ref: 15–34)		
**35**–**49**	0.82 (0.78, 0.87)	<0.001
**50 and above**	0.79 (0.74, 0.84)	<0.001
**Gender of house head** (ref: Male)		
Female	0.83 (0.76, 0.9)	<0.001
**Education of house head** (ref: None or pre-primary)		
Primary	0.91 (0.86, 0.97)	<0.001
Secondary	0.88 (0.82, 0.94)	0.003
Higher secondary/above	0.77 (0.69, 0.85)	<0.001
**Media exposure** (ref: No)		
Yes	0.83 (0.78, 0.87)	<0.001
**Mobile phone ownership** (ref: No)		
Yes	1 (0.95, 1.05)	0.917

AOR: Adjusted odds ratio; CI: Confidence interval.

## Data Availability

This article does not contain any studies with human participants performed by any of the authors. Data used in research were attained from Multiple Indicator Survey (MICS), which was carried out by Bangladesh Bureau of Statistics (BBS) in collaboration with UNICEF Bangladesh, as part of the Global MICS Programme. All identification of the respondents was dis-identified before publishing the data. The secondary data sets of the current study are available at http://mics.unicef.org/tools?round=mics6 (accessed: 10 December 2021). Permission for this project was taken from the MICS Program authority by the authors.
